# Developing and evaluating an educational intervention on conflicts of interest and corporate influence on science

**DOI:** 10.1093/heapro/daaf059

**Published:** 2025-05-22

**Authors:** Alice Fabbri, Iona Fitzpatrick, Sophie Braznell, Tess Legg, Emma Cliffe, Filipa Vance, Dale Topley, Fran Baber, Anna B Gilmore

**Affiliations:** Department for Health, University of Bath, Claverton Down, Bath BA2 7AY, United Kingdom; Department for Health, University of Bath, Claverton Down, Bath BA2 7AY, United Kingdom; Department for Health, University of Bath, Claverton Down, Bath BA2 7AY, United Kingdom; Department for Health, University of Bath, Claverton Down, Bath BA2 7AY, United Kingdom; Mathematics Resources Centre, University of Bath, Claverton Down, Bath BA2 7AY, United Kingdom; Research Governance and Compliance, University of Bath, Claverton Down, Bath BA2 7AY, United Kingdom; Research Governance and Compliance, University of Bath, Claverton Down, Bath BA2 7AY, United Kingdom; Research Governance and Compliance, University of Bath, Claverton Down, Bath BA2 7AY, United Kingdom; Department for Health, University of Bath, Claverton Down, Bath BA2 7AY, United Kingdom

**Keywords:** conflicts of interest, research integrity, commercial determinants of health, educational intervention, training

## Abstract

Financial conflicts of interest resulting from corporate funding of research can bias the evidence base. We designed an educational intervention that sought to enable participants to make informed decisions and mitigate risk when considering corporate funding for research. We used pre/post-test surveys, which comprised a mix of closed and open-ended questions, to evaluate the training and its impact on knowledge (Wilcoxon signed-rank test), attitudes and perceptions (Friedman’s test with planned post hoc tests). Open-ended questions were coded and key themes identified. Twenty participants from the University of Bath (15 PhD students and 5 research staff) completed the pre-test survey and attended the training, 17 filled in the post-test survey, and 17 filled in the 3-month follow-up survey. All participants agreed or strongly agreed that the issues relating to conflict of interest presented in the training increased their interest in the topic. Participants’ knowledge significantly increased between the pre and post-measures. Awareness of institutional conflict of interest policies and participants’ confidence in mitigating the risks of corporate funding also significantly improved. For the other measures of impact, either there was not a statistically significant difference between the pre, post, and follow-up measures or there was, but post hoc tests were not significant after a Bonferroni correction. Our findings indicate that even a short educational intervention could increase researchers’ confidence in and ability to make informed decisions about whether to accept corporate funding and under what conditions.

Contribution to Health Promotion

- Financial conflicts of interest resulting from corporate funding of science can have a significant impact on research integrity. We designed, delivered, and evaluated an educational intervention on conflicts of interest for PhD students and research staff at the University of Bath.
- The training content and delivery were generally well received. Participants’ knowledge of the course topic significantly increased as well as participants’ confidence in knowing how to mitigate the risks of corporate funding.
- Even a short educational intervention could be effective in empowering individuals to make informed decisions about whether to accept corporate funding and how to navigate those funding relationships.

## INTRODUCTION

In recent decades, corporations have become increasingly prominent in research funding (Moses et al. 2015). Corporate funding of research has helped advance many branches of science, but evidence has shown that in some cases, financial conflicts of interest arising from corporate funding can also have a significant negative impact on the scientific process (Thomas et al. 2025). A recently published typology of corporate influence on science has shown that corporations recurrently use the same set of strategies to influence the whole scientific process from evidence production to the use of science in policy and practice in ways that systematically favour their interests (Legg et al. 2021). When it comes to evidence production corporations have been shown to influence every step, starting from the ways in which research questions are framed (Fabbri et al. 2018, Legg et al. 2021). For example, internal industry documents from 1959 to 1971 revealed that the sugar industry successfully diverted attention from interventions aimed at reducing sugar intake by funding research into a vaccine against dental caries and into enzymes that break up dental plaque (Kearns et al. 2015). The following steps of the research process—the design, conduct, and reporting of findings, can also be influenced by corporate funders (Bero 2019). For example, pharmaceutical companies have misrepresented or concealed the results of clinical trials on their products, thus distorting the evidence base that physicians use to make prescribing decisions (Avorn 2006).

By influencing science, corporations are able to produce and promote evidence highlighting the benefits and obscuring the harms of their products. This can in turn, undermine and delay the adoption and effective implementation of policies that threaten corporate profitability, leading to millions of avoidable deaths—tobacco and climate change being key examples—and protect corporations against litigation seeking to hold them to account for the harm they have caused (Legg et al. 2021).

Corporate funding and subsequent financial conflicts of interest can also pose significant risks to academic institutions and individual researchers. The conduct of academic institutions is subject to significant public scrutiny, with many openly declaring their commitments to responsible research and high standards of rigour as signatories to The Concordat to Support Research Integrity (Universities UK 2019). Corporate funding can create potential for the misuse of University resources and research in order to further corporate interests in ways contrary to the University’s mission (Charles Perkins Centre 2016), which can contribute to the undermining of public trust in science.

For individual researchers, corporate funding can lead to reputational harm and loss of future opportunities due to negative perceptions of industry connections (Mitchell and McCambridge 2021). Although researchers often view themselves as objective creators of science and feel that their training as scientists will protect them from external influences, evidence shows that financial relationships impose a sense of indebtedness on the recipient that can have an impact on behaviour, whether the recipient is conscious of it or not (Katz et al. 2003).

The forces at play are complex, and existing research suggests that institutional resources do not adequately empower researchers to manage them (Mumford et al. 2008). Moreover, an analysis of UK Universities’ policies concerning funding from health-harming industries has exposed a lack of institutional guidance for managing those relationships (Collin et al. 2021).

We therefore developed an educational intervention aiming to improve researcher confidence to make informed decisions and better mitigate risk when considering corporate research funding. The aims of this study were to:

- develop and pilot an educational intervention focussed specifically on financial conflicts of interest and corporate influence on science for PhD students and research staff at the University of Bath.
- evaluate the training and its impact on knowledge, skills, attitudes, and behaviours of researchers (staff and students).

## MATERIALS AND METHODS

We capitalized on the specialist expertise relating to corporate influence on science of the research team and collaborated with members of the University of Bath’s Research Governance and Compliance team (RG&C) whose remit includes the provision of training in research governance, ethics, and integrity to design, deliver and evaluate a 2.5-h online training session.

The training session included the presentation of published evidence on how corporations have attempted to influence science and its use in policy and practice (Legg et al. 2021). Using the ‘cycle of bias’ framework (Bero 2023), we showed participants how corporate funding can influence each step of the research process (Fabbri et al. 2018, Legg et al. 2021). We illustrated how partnerships between industry and academia can create conflicts of interest and the risks and implications of these conflicts. We then gave participants an overview of potential solutions to the problem: from short-term solutions (e.g. improving the transparency of financial interest and developing clear conflict of interest policies for research institutes) to long-term solutions (e.g. funding independent research through dedicated manufacturer taxes or legally mandated contributions) (Fabbri and Gilmore 2023). Finally, we provided participants with a self-assessment tool for them to use when they are considering entering into a research partnership with a corporation and gave an overview of the University of Bath policies and procedures for managing risks when interacting with corporations.

The training included two interactive breakout sessions where participants were separated into groups and given fictionalized proposals for a research project with an external partner. The proposals were drawn from real examples of project agreements and experiences involving industry-funded science supplied by academics working in related fields. One of the project team acted as a moderator in the breakout sessions. Participants were asked to discuss the proposals, identify potential concerns, and decide whether the research collaboration should be permitted or altered before agreement (see Supplementary File 1 for the training program).

The optional, stand-alone training was advertised to research staff and PhD students at the University of Bath between July and September 2023 via multiple routes such as Twitter, Yammer, newsletters, emails and at an internal event where some of the researchers were in attendance (word of mouth). The training took place in September 2023.

Using a pre/post-test survey evaluative approach, we collected data on departmental affiliation, previous training in conflict of interest and measures to assess the impact of the training on participants’ knowledge attitudes and behaviours (Supplementary File 2). We also asked participants to rate themselves on how left- or right-wing they considered themselves, since previous research has demonstrated that worldview can affect individuals’ perceptions of both science and industry involvement in science (Lewandowsky and Oberauer 2021, Legg et al. 2024). The pre-test was sent to participants before the course, the post-test at the end of the course, and a follow-up survey three months later. The surveys included both closed and open-ended questions; closed questions were a mix of Likert Scales, checklist and dichotomous multiple-choice questions. Open-ended questions were included with the intention of gathering contextual insight regarding researcher experience of financial conflicts of interests and feedback to inform the design of future training. Participants could choose not to answer any question by not providing a response.

### Analysis

#### Quantitative analysis

The repeated ordinal Likert items measuring aspects of attitude and perception were compared via the non-parametric Friedman’s test with planned post hoc tests comparing pre and post-measures and pre and follow-up measures. These post hoc tests are non-parametric Wilcoxon signed-rank tests with a Bonferroni correction; when the assumption of symmetric differences was violated the sign test, with Bonferroni correction, was used. Power analysis for the post hoc Wilcoxon signed-rank tests suggested a 73% chance of detecting a large effect (achieved sample size, α = 0.025, *d* = 0.8). No power analysis for Friedman’s tests is reported as the assumptions for available methods of calculation did not hold. The knowledge before and after the training was compared via the Wilcoxon signed-rank test. Power analysis suggested an 83% chance of detecting a large effect (achieved sample size, *α* = 0.05, *d* = 0.8).

We had also originally planned to explore the relationships between the pre-post/follow-up attitude, perception and knowledge change measures and demographic/experience measures to generate hypotheses regarding interactions but were unable to run these analyses as we did not achieve a sufficient sample size.

#### Adjunctive analysis of open-ended responses

Our analysis of the open-ended response data was a hybrid method of thematic analysis, adapted from two studies centred on the identification of themes in qualitative data (Robinson 2022, Naeem et al. 2023), based on the requirements of this pilot study. Once all pre-, post- and follow-up survey responses had been received, open-ended response data were compiled (each having their own row) in a spreadsheet in line with the structured approach recommended by Robinson when dealing with ‘brief texts’ (Robinson 2022).

All responses were read through in their entirety by IF. Following Naeem et al. (2023), themes were developed in three stages: (i) keywords were identified in individual responses; (ii) topics were developed based on repeated keywords, and (iii) overarching themes were developed. The entire data set was then independently coded to these themes by both IF and SB. Responses could be coded to multiple themes. In line with Robinson, we then checked inter-analyst agreement between IF and SB coding and explored the frequencies of each theme.

A post-coding discussion between SB and IF considered the sufficiency of the overarching framework and enabled the refinement of theme descriptors. We report illustrative examples from the overarching themes under results and have included frequency reporting of coded themes following Robinson’s argument that they ‘convey some information on the salience and importance of a theme to the study’s message’ (Robinson 2022).

## RESULTS

### Participants’ characteristics

After excluding four participants (two did not fully fill in the consent form, one did not meet the inclusion criteria, and one left the training within the first 30 min), 20 participants both completed the pre-test survey and attended the training, 17 filled in the post-test survey and 17 filled in the follow-up survey. However, only 16 participants completed the pre, post-test, and follow-up surveys. In the follow-up survey two participants mentioned their job title or institutional affiliation had changed since they attended the training; we decided to still include them in the analysis.

Table 1 shows the participants’ characteristics at baseline. Of the 20 participants, 15 were PhD students and 5 were research staff. They had a median research experience of 4.5 years but with a range of 29 years. Only two participants mentioned they had received prior training on financial conflicts of interest in research.

**Table 1.** Participants’ characteristics at baseline (*n* = 20).

Participants’ characteristics	*n*
Gender	Female = 13 Male = 6 Other = 1
Age range	18–24 = 1 25–35 = 10 36–45 = 5 46–55 = 2 65+=2
Position	PhD student = 15 Postdoctoral researcher = 4 Professor = 1
Department	Health = 14 Architecture and Civil Engineering = 2 Life sciences = 1 Mathematics = 1 Psychology = 1 Social and Policy Sciences = 1
Political views scored on a scale between 0 to 10, with 0 being most ‘left-wing’ and 10 being most ‘right-wing’	0: *n* = 1 1: *n* = 1 2: *n* = 2 3: *n* = 5 4: *n* = 4 5: *n* = 2 6: *n* = 1 Don’t know: *n* = 4
Have received training on financial conflicts of interest in research such as commercial sponsorship of research at the University of Bath	Yes = 1 No = 19
Have received external training on financial conflicts of interest in research such as commercial sponsorship of research	Yes = 1 No = 19
Have received corporate funding for my research^a^	Yes = 4 No = 16
Those I work with/for have received corporate funding for research in which I have been involved^*^	Yes = 5 No = 11 Do not know = 4

^a^Pharmaceuticals were mentioned by three participants, biotechnology, heavy manufacturing (steel) and food were each mentioned once.

### Keywords, topics, and themes in open-ended responses

The analysis of open-ended responses generated a list of 14 topics (advantages, ambition, collaboration, colleagues, expertise, hierarchy, imbalance, models, money, opportunity, personal ethics, political leaning, time, university systems), which was refined to a framework of the following themes: Power, Resources, Environment, Drive and Network. This framework integrates feedback relating to both the operational and theoretical facilitators and inhibitors in the participants’ experience of training and their ability to enact change in their own practice relating to financial conflicts of interest. Supplementary File 3 shows a few examples of the development of themes from text to theme.

We have reported the number of responses coded to each open-ended question throughout the following section, alongside illustrative examples.

### Participants’ experiences and expectations (pre-test survey)

Six participants indicated either they or the people that they work with or for had received funding from corporate funders, these corporate sectors are listed in Table 1. Four participants reported some resource-related positive aspects of corporate funding, including adequate financial support (4/6) and access to expertise (3/6):

*“Funds allowed us to conduct research we would not have been able to do otherwise.”*

The corporate funder’s involvement in subject/study area selection was mentioned by three participants (3/6) and one of these also mentioned funder involvement in study design and decision to publish/not publish. When asked about limitations on publishing results, seven people answered. The ability of the funder to control the outputs (3/7) was noted as a limiting factor. In some cases, the exact contribution of the funder to the research project was not clear.

*“There is a feeling that I should be producing something of value to the company. I worry that I take their funding help somewhat for-granted and am unsure what the balance is as to their involvement in the project.”*

Participants were also asked to clarify their expectations for the intervention. Concerns with imbalance in relationships and influence (positive or negative) were expressed by all but five of the participants (15/20).

*“I would like to be able to know how to mitigate influence without damaging relationships or research integrity.”*

Seeking information and guidance was a key motivating factor for a majority of participants (14/20); a desire to understand and mitigate risk was also raised by several participants (5/20).

*“I am concerned that there are issues that I am not aware of. What do I do if I embark on funded research and subsequently realize there is a COI (sic).”*

### Evaluation of the training

The training content and delivery were generally well received with all the participants agreeing/strongly agreeing that the problems around conflict of interest presented in the training increased their interest in the topic (Table 2).

**Table 2.** Training evaluation (*n* = 17).

	Strongly agree	Agree	Neither agree nor disagree	Disagree	Strongly disagree
The problems around conflict of interest presented in the training increased my interest in the course topic	8	9	0	0	0
Course goals were clearly communicated	10	6	0	1	0
The instructors were helpful in guiding the class towards understanding course topics	12	5	0	0	0
I felt comfortable participating in the discussions	10	6	0	1	0

In the post-test survey, we also asked participants to rate how relevant the course content was to their current work: 5/17 rated it as extremely relevant, 6/17 as very relevant, 5/17 as moderately relevant, and one participant did not reply. We also asked whether they would use what they learned in the course in their work: 9/17 said ‘definitely yes’, 7/17 ‘probably yes’ and one said they did not learn anything new.

When asked specifically about possible improvements to the training, one participant did not provide a response but the majority suggested alterations to the timing (11/17). Some (4/17) felt that the training could have been extended or broken into several sessions. A few participants (3/17) stressed the value of the training in leveraging existing resources, specifically facilitating networking and learning from colleagues.

*“It would have been good to have more time in the breakout rooms, as participants had very interesting experiences with this topic and as it was we did not have enough time to discuss these.”*

The parts of the course that were most helpful to participant learning concerned resource availability; a dedicated space to discuss case studies and the expertise of colleagues (7/16).

In the post-test survey in response to ‘anything to add?’ (12/17), a majority (8/12) expressed positive feelings and several people suggested extending either the scope of the material covered in the session (4/12), or the audience to which the session was offered (1/12).

In the follow-up survey, in response to ‘anything to add?’ this majority held, with five (5/6) participants expressing positive sentiment regarding the value of the training. Half (3/6) of the suggestions for change concerned extending the offering, for either audience or content. One participant suggested that the training could be more valuable in contexts where corporate funding was commonly encountered.

*“In my area of work, accepting funding from corporations is seen as highly unethical and so I feel as though the training only reinforced those norms. It would probably benefit researchers, especially ECRs, in other areas where corporate funding is more widespread.”*

All participants in the follow-up survey stated they would recommend the training to colleagues or students: 10/17 strongly agreed and 7/17 agreed.

### Impact of the training

#### Knowledge, attitudes, and behaviours

Participants’ knowledge of the course topic was measured at pre [median Interquartile range (IQR) = 2.00 (2.00–3.00)] and post [median (IQR) = 4.00 (3.00–5.00)] time points. There was a significant increase, with a large effect size, between the pre and post-measures (*Z* = −3.442, *P* < 0.001, *r* = 0.861).

As Table 3 shows, we found a statistically significant difference in how confident participants felt in mitigating the risks of corporate funding. Post hoc tests suggested that there was a significant increase in confidence, with a large effect size, immediately post-course and that this persisted at follow-up.

**Table 3.** Pre/post/follow-up change.

Question^a^	Pre-test Median (IQR)	Post-test Median (IQR)	Follow-up Median (IQR)	Friedman X^2^, *P*-value, effect size (W)	Pre/post post hoc test, effect size (*r*)	Pre/follow-up post hoc test, effect size (*r*)
I am likely to accept corporate funding for my research in the future	3.00 (1.25–4.00)	2.00 (1.00–4.00)	3.00 (1.00–4.00)	*Χ* ^2^ (2) = 7.000, *P* = .030 Small (*W* = 0.219)	*Z* = −2.126, *P* = .033 Large (*r* = 0.532)	Z = −2.121, *P* = .034 Large (*r* = 0.530)
Corporate funding of research is essential to the future of research	2.50 (2.00–3.75)	3.00 (1.25–4.00)	2.00 (1.00–3.00)	*Χ* ^2^ (2) = 4.044, *P* = .132 Small (*W* = 0.126)		
Corporate funding of academic research can create a conflict of interest	5.00 (4.00–5.00)	5.00 (4.00–5.00)	5.00 (4.25–5.00)	*Χ* ^2^ (2) = 1.182, *P* = .554 Negligible (*W* = 0.037)		
The risks to researchers of accepting funding from corporations are exaggerated	2.00 (2.00–3.00)	2.00 (1.00–2.00)	2.00 (1.25–2.00)	*Χ* ^2^ (2) = 6.545, *P* = .038 Small (*W* = 0.205)	*Z* = −1.941, *P* = .052 Moderate (*r* = 0.485)	*Z* = −2.111, *P* = .035 Large (*r* = 0.528)
Corporate funding of academic research can create risks of undue influence on the research process	4.00 (4.00–5.00)	5.00 (4.00–5.00)	4.50 (4.00–5.00)	*Χ* ^2^ (2) = 1.143, *P* = .565 Negligible (*W* = 0.036)		
Corporate funding of academic research can compromise academic freedom	4.00 (3.00–5.00)	5.00 (4.00–5.00)	4.50 (4.00–5.00)	*Χ* ^2^ (2) = 7.032, *P* = .030 Small (*W* = 0.220)	*Z* = −1.999, *P* = 0.046 Large (*r* = 0.500)	*Z* = −2.111, *P* = .035 Large (*r* = 0.528)
Universities should develop policies to protect research integrity from corporate influence	5.00 (4.25–5.00)	5.00 (5.00–5.00)	5.00 (5.00–5.00)	*Χ* ^2^ (2) = 6.500, p = 0.039 Small (*W* = 0.203)	*P* = .125^b^	*P* = 1.000^b^
I am aware of the policies and procedure the University of Bath has in place to monitor and manage conflicts of interest in research	2.00 (2.00–4.00)	4.00 (4.00–4.75)	4.00 (3.00–4.00)	*Χ* ^2^ (2) = 15.273, *P* < .001 Moderate-large (*W* = 0.477)	*Z* = −3.140, *P* = .002 Large (*r* = 0.785)	*Z* = −2.204, *P* = .027 Large (*r* = 0.551)
I would know how to manage the risks of corporate funding	3.50 (2.00–4.00)	4.00 (4.00–4.00)	4.00 (3.25–4.00)	*Χ* ^2^ (2) = 2.2053, *P* = .358 Negligible (*W* = 0.069)		
I feel confident that I know how to mitigate the risks of corporate funding	2.50 (2.00–3.00)	4.00 (4.00–4.00)	4.00 (3.25–4.00)	*Χ* ^2^ (2) = 16.146, *P* < .001 Large (*W* = 0.505)	*Z* = −3.017, *P* = .003 Large (*r* = 0.754)	*Z* = −2.570, *P* = .010 Large (*r* = 0.643)

^a^Possible answers to these questions were: 1 (Strongly disagree), 2 (Disagree), 3 (Neither agree nor disagree), 4 (Agree), 5 (Strongly agree); exact wordings and coding are provided in Supplementary File 2.

^b^The assumptions of the post hoc Wilcoxon signed-rank test were not met and so the sign test, which has less power, was used.

There was also a statistically significant difference in awareness of the University of Bath policies and procedure to monitor and manage conflicts of interest in research. Post hoc tests suggested that there was a significant improvement in awareness, with a large effect size, immediately post-course but it is unclear whether this persists (Table 3).

For the other questions reported in Table 3 either we did not find a statistically significant difference within the pre, post, and follow-up measures (Friedman test) or we did find it but the post hoc tests were not significant after a Bonferroni correction.

Fourteen participants either strongly agreed (8/14) or agreed (6/14) that the training had a positive impact on their research practices. When asked about what (if anything) they planned to use from the course, a majority of participants (9/14) mentioned a change in attitude or intended behaviour as a result of the training. Half of participants (7/14) stated an intention to use the resources provided during the training to aid them in navigating researcher-funder relationships.

*“Knowledge about how to identify ‘red flags’ in invitations to collaborate with external partners and how to assess whether the partnership aligns with my morals and values as a researcher”.*

*‘I also plan to use the course guidance to assist me in identifying conflicts of interest and to establish who maybe affected by them. Lastly, if I am approached to collaborate with industry/researchers with industry funding, I will refer back to the guidance provided within this course to make an informed decision’.*

Ten participants either agreed (7/17) or strongly agreed (3/17) with the statement ‘I have applied the knowledge created in this course to my work’. Twelve agreed (9/17) or strongly agreed (3/17) with the statement ‘I have applied the tools learned during this course in practice’. In the follow-up survey, four participants (4/17) did not provide a response to the question about planned or enacted change. Only three participants reported that they had taken action since the training (3/13).

*“I have talked to colleagues about the issues”*

*“I was able to apply the information on areas that might compromise the researcher/research. This included keeping the research question open so it didn’t lead the direction of the review, maintaining researcher independence and integrity and asking to demonstrate that they were independent and provide plans to mitigate potential bias/influence from corporate stakeholders. “*

#### Perceived supporting and limiting factors

When asked about the most important factor limiting application of content learned during the course, participants most commonly noted environmental factors (13/17), either a perceived lack of support from the University (6/17) and/or the lack of opportunity to apply their skills in a practical setting (8/17).

Support from supervisors or colleagues was also viewed as a key limiting factor (7/17).

*“I’ve tried to apply the learnings but my supervisor doesn’t seem to have the same concerns I have learned regarding conflict of interest”*

Fifteen participants reported enabling factors; particularly the opportunity to apply the learning in a practical setting (6/15) and support from colleagues (4/15) or supervisors (4/15). Support from the University as positive was only selected by two participants (2/15).

## DISCUSSION

We developed and piloted a novel educational intervention on conflicts of interest and corporate influence on science for PhD students and research staff at the University of Bath. These topics appear to be a neglected area as only two participants mentioned they had received prior training, despite a median research tenure of 4.5 years. Overall, the training was positively received. Knowledge of the course topic improved following the training, suggesting that even a short educational intervention can have a positive impact on researchers’ understanding of these issues. Despite the small sample size limiting our analyses of impact on attitudes and behaviours, participants’ awareness of policies and confidence in knowing how to mitigate the risks of corporate funding significantly improved following the training. In general, the reported change in outcomes was modest. In this regard, it is important to mention that the intervention and surveys were conducted on a small sample that was self-selecting and may over-represent researchers with a higher level of interest and background knowledge than in any wider population.

The analysis of the open questions demonstrates that the training generated at least a self-reported intent in researchers to change their behaviour, whether in the form of increased vigilance or a desire to start having conversations about funding with colleagues and collaborators. Positive sentiments towards the intervention were also long-lasting, with a majority of participants providing positive statements about the intervention three months later in the follow-up survey.

There was a complex interplay of themes in the narrative response data. Power and resources were closely linked and were commonly co-coded. Money, time and practical opportunity to apply learnings all intersected with the reported ability of the participants to enact change. Researchers were keenly aware that imbalances of power in their professional networks (e.g. between researchers at different career stages) impacted their working lives. Participants expressed an understanding that funding relationships are based on mutual gains, but it was clear that this reciprocity is not always well balanced. Four participants expressed the sentiment that corporate funding was a necessary factor in the facilitation of some types of research and that without corporate funding, some research would not happen at all. Sentiments relating to power were closely connected to mentions of resources—time, salary and equipment. Both time and money were framed as scarce by participants. Corporate funding offered the opportunity to have more through access to a wider network of expertise and the provision of research equipment.

### Limitations

The intervention and surveys were conducted on a small sample that was self-selecting and is unlikely to be representative of the University of Bath population of research staff and students, or of any larger population of academic researchers. However, this analysis was exploratory with the primary aim, not of making inferences to any wider population, but of generating insights for future research and interventions. Moreover, participants were predominantly from the department of health, possibly due to familiarity bias arising from departmental affiliation of five of the authors. In some research areas like public health, the tensions between corporate interests and disciplinary aims can be more readily apparent and are therefore more prominent in the day-to-day work of those research groups.

Since there is not currently a validated questionnaire to evaluate a training on conflict of interest in research, we developed our own surveys, which were reviewed internally for face validity. By using a non-validated survey there is a possibility that bias was introduced through phrasing, given the shared research areas of several of the authors who were involved in the study design.

Open-ended questions had low response rates; in a future study, this could be overcome by altering survey design (i.e. mandating responses). The data volume generated by the open-ended questions did not permit an open and organic, reflexive approach to thematic coding. Most open-response data only made sense within the frame of the question. A future study might seek to draw out richer insights through the use of interviews or focus groups following the educational intervention or through the alteration of questionnaire design to enable greater participant-led reflection.

### Lessons learned and implications

The fact that most of our participants were PhD students possibly indicates that they are eager to learn more about these topics and may be a good target for these types of educational interventions. Being more sensitive to University timelines for a variety of research areas might also help to improve turnout, as some research areas or career stages face different work burdens at different times of the year.

Research has highlighted that often research integrity and ethics training courses provide only basic guidelines and rules and do not equip researchers with the tools demanded by complex, real-world situations (Mumford et al. 2008). Moreover, researchers are not uniquely burdened by conflicts of interests (Fabbri and Grundy 2024). Our intervention was designed to capitalize on the existing experiences of participants and included case studies that involved collaborative learning. Participants expressed positive feelings about the breakout sessions and hearing about the experiences of their peers, including the trainers. However, several participants felt the sessions could have better supported this element by being longer. Further interventions should capitalize on this existing enthusiasm and be sure to include as much participant interaction and discussion as possible.

Trainers should be mindful of institutional and social structures that may inhibit the exchange of information or the application of learnings, as some of our participants reported (Billett 2022). Our work supports arguments that highlight the complex interplay of systems and individuals and suggests that learning must be facilitated at multiple levels in order to overcome barriers to effective learning (Okpalauwaekwe et al. 2024). This would likely mean supporting researcher development through multiple channels rather than through simple resource provision, or one-off trainings. Finally, actions to protect research integrity should be multi-faceted; beyond training they should include the development of robust institutional conflict of interest policies and due diligence processes.

## CONCLUSION

Training on how to manage conflicts of interest in science is a neglected area. Our findings indicate that even a short educational intervention could drive change by increasing researcher confidence and empowering individuals to make informed decisions about whether to accept corporate funding and how to negotiate those relationships.

## Supplementary Material

daaf059_suppl_Supplementary_File_S1

daaf059_suppl_Supplementary_File_S2

daaf059_suppl_Supplementary_File_S3

## Data Availability

The data underlying this article are available in the article and in its online supplementary material.
