# Supplementary material for: Developing and evaluating an educational intervention on conflicts of interest and corporate influence on science
Source: Health Promot Int. 2025 May 22;40(3):daaf059. doi: 10.1093/heapro/daaf059 (PMC12096445; doi:10.1093/heapro/daaf059)
Supplement: daaf059_suppl_Supplementary_File_S1 [file daaf059_suppl_supplementary_file_s1.docx]

**Supplementary File 1. Program**

**Developing and evaluating an educational intervention to improve research integrity by addressing conflicts of interest and corporate influence on science**

| 10 – 10.15 | Welcome | |
| --- | --- | --- |
| 10.15-10.30 | Interactive session (part 1) | |
| 10.30-10.50 | 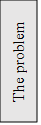 | The Science for Profit Model |
| 10.50-11.00 |  | Conflicts of interest (definitions, how they arise) |
| 11.00-11.10 |  | Implications and risks |
| 11.10-11.15 | Q&A | |
| Break (5 minutes) | | |
| 11.20-11.35 | 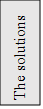 | Solutions to prevent/manage conflicts of interest |
| 11.35-11.50 |  | University of Bath’s procedures to prevent/manage conflicts of interest |
| 11.50-11.55 | Q&A | |
| 11.55-12.10 | Interactive session (part 2) | |
| 12.10-12.25 | Feedback and discussion | |
| 12.25-12.30 | Closing remarks | |
