# Supplementary material for: Developing and evaluating an educational intervention on conflicts of interest and corporate influence on science
Source: Health Promot Int. 2025 May 22;40(3):daaf059. doi: 10.1093/heapro/daaf059 (PMC12096445; doi:10.1093/heapro/daaf059)
Supplement: daaf059_suppl_Supplementary_File_S2 [file daaf059_suppl_supplementary_file_s2.docx]

**Supplementary File 2. Surveys**

**Pre-test**

Participants’ characteristics:

- Department
- Job title: PhD student, postdoc, lecturer, senior lecturer, professor, other. Teaching staff will be excluded.
- How would you describe your gender? Female , Male, Other, Prefer not to say

If you chose other, please describe here if you feel comfortable doing so.

- Age: 18-24, 25-35, 36-45, 46-55, 56-65, 65+
- How many years of research experience do you have?
- People sometimes use the labels 'left' or 'left-wing' and 'right' or 'right-wing' to describe political parties, party leaders, and political ideas. Using a 0 to 10 scale, where 0 means left and 10 means right, where would you place yourself on this scale? (Note: we also added a ‘Don’t know’ option).

Attitudes and perceptions

- I am likely to accept corporate funding for my research in the future (1=Strongly disagree, 2=Disagree, 3=Neither agree nor disagree, 4=Agree, 5=Strongly agree)
- Corporate funding of research is essential to the future of research (1=Strongly disagree, 2=Disagree, 3=Neither agree nor disagree, 4=Agree, 5=Strongly agree)
- Corporate funding of academic research can create a conflict of interest (1=Strongly disagree, 2=Disagree, 3=Neither agree nor disagree, 4=Agree, 5=Strongly agree)
- The risks to researchers of accepting funding from corporations are exaggerated (1=Strongly disagree, 2=Disagree, 3=Neither agree nor disagree, 4=Agree, 5=Strongly agree)
- Corporate funding of academic research can create risks of undue influence on the research process (1=Strongly disagree, 2=Disagree, 3=Neither agree nor disagree, 4=Agree, 5=Strongly agree)
- Corporate funding of academic research can compromise academic freedom (1=Strongly disagree, 2=Disagree, 3=Neither agree nor disagree, 4=Agree, 5=Strongly agree)
- Universities should develop policies to protect research integrity from corporate influence (1=Strongly disagree, 2=Disagree, 3=Neither agree nor disagree, 4=Agree, 5=Strongly agree)
- I am aware of the policies and procedure the University of Bath has in place to monitor and manage conflicts of interest in research (1=Strongly disagree, 2=Disagree, 3=Neither agree nor disagree, 4=Agree, 5=Strongly agree)
- I have received training on financial conflicts of interest in research such as commercial sponsorship of research at the University of Bath (Yes/No)
- I have received external training on financial conflicts of interest in research such as commercial sponsorship of research (Yes/No)
- If yes, please specify (where, how long)
- I would know how to manage the risks of corporate funding (1=Strongly disagree, 2=Disagree, 3=Neither agree nor disagree, 4=Agree, 5=Strongly agree)
- I feel confident that I know how to mitigate the risks of corporate funding (1=Strongly disagree, 2=Disagree, 3=Neither agree nor disagree, 4=Agree, 5=Strongly agree)

Upcoming training

- Open question: the upcoming training will focus on conflicts of interest and corporate influence on science. Could you please tell us what you would like to get out of this training? (Open question)
- Rate your knowledge of the course topic (1=Not at all knowledgeable, 2=Slightly knowledgeable, 3=Moderately knowledgeable, 4=Very knowledgeable, 5=Extremely knowledgeable)

Experiences

- I have received corporate funding for my research (Yes/No)
- Those I work with/for have received corporate funding for research in which I have been involved (Yes/No/Don’t know) (Note: you can say yes if the funding did not go directly to you, but to your boss or a colleague)
- If you replied “Yes” to the previous two questions, please answer the following questions. If you answered "No", you can submit the survey.
  - Please list from which corporate sectors you have received funding (e.g. food, alcohol, gambling, chemical, fossil fuel, pharma, medical device industry other (please specify))
  - Please describe any benefits to your collaboration
  - Please describe any negative aspects of the collaboration
  - Was the corporate sponsor involved in: (tick any that apply)
    - Choice of subject/study area
    - Study design
    - Data analysis
    - Writing of the manuscript
    - Decision to publish/not publish
    - Dissemination
    - None of the above
  - Did you have freedom to publish?

**Post test**

Course assessment

- Course goals were clearly communicated (1=Strongly disagree, 2=Disagree, 3=Neither agree nor disagree, 4=Agree, 5=Strongly agree)
- The instructors were helpful in guiding the class towards understanding course topics (1=Strongly disagree, 2=Disagree, 3=Neither agree nor disagree, 4=Agree, 5=Strongly agree)
- I felt comfortable participating in the discussions. (1=Strongly disagree, 2=Disagree, 3=Neither agree nor disagree, 4=Agree, 5=Strongly agree)
- The problems around conflict of interest presented in the training increased my interest in the course topic. (1=Strongly disagree, 2=Disagree, 3=Neither agree nor disagree, 4=Agree, 5=Strongly agree)
- Rate your knowledge of the course topic (after attending the training) (1=Not at all knowledgeable, 2=Slightly knowledgeable, 3=Moderately knowledgeable, 4=Very knowledgeable, 5=Extremely knowledgeable)
- How relevant is this course to your current work? (1=Not at all relevant, 2=Slightly relevant, 3=Moderately relevant, 4=Very relevant, 5=Extremely relevant)
- Will you use what you learned in this course in your work? (Definitely not, Probably not, Possibly, Probably yes, Definitely yes, I did not learn anything new from this course)
- What, if anything, do you plan to use from this course? (open-ended)
- Which of these factors would **most** impact your ability to apply what you have learnt on this course? (Select all that apply: Time, support from colleagues, support from my supervisor, support from the University, opportunity to apply skills in a practical setting, Other (please specify))
- How could this course be improved to make it a more effective learning experience? (open-ended)
- What part of this course was most helpful to your learning? (open-ended)
- Is there anything you would like to add?

| Attitudes and perceptions   - I am likely to accept corporate funding for my research in the future (1=Strongly disagree, 2=Disagree, 3=Neither agree nor disagree, 4=Agree, 5=Strongly agree) - Corporate funding of research is essential to the future of research (1=Strongly disagree, 2=Disagree, 3=Neither agree nor disagree, 4=Agree, 5=Strongly agree) - Corporate funding of academic research can create a conflict of interest (1=Strongly disagree, 2=Disagree, 3=Neither agree nor disagree, 4=Agree, 5=Strongly agree) - The risks to researchers of accepting funding from corporations are exaggerated (1=Strongly disagree, 2=Disagree, 3=Neither agree nor disagree, 4=Agree, 5=Strongly agree) - Corporate funding of academic research can create risks of undue influence on the research process (1=Strongly disagree, 2=Disagree, 3=Neither agree nor disagree, 4=Agree, 5=Strongly agree) - Corporate funding of academic research can compromise academic freedom (1=Strongly disagree, 2=Disagree, 3=Neither agree nor disagree, 4=Agree, 5=Strongly agree) - Universities should develop policies to protect research integrity from corporate influence (1=Strongly disagree, 2=Disagree, 3=Neither agree nor disagree, 4=Agree, 5=Strongly agree) - I am aware of the policies and procedure the University of Bath has in place to monitor and manage conflicts of interest in research (1=Strongly disagree, 2=Disagree, 3=Neither agree nor disagree, 4=Agree, 5=Strongly agree) - I would know how to manage the risks of corporate funding (1=Strongly disagree, 2=Disagree, 3=Neither agree nor disagree, 4=Agree, 5=Strongly agree) - I feel confident that I know how to mitigate the risks of corporate funding (1=Strongly disagree, 2=Disagree, 3=Neither agree nor disagree, 4=Agree, 5=Strongly agree)   **Follow-up**   - Has your job title or institutional affiliation changed since you attended the training in September 2023? (e.g. you completed the PhD, you moved to another institution) - If yes, please specify   Attitudes and perceptions   - I am likely to accept corporate funding for my research in the future (1=Strongly disagree, 2=Disagree, 3=Neither agree nor disagree, 4=Agree, 5=Strongly agree) - Corporate funding of research is essential to the future of research (1=Strongly disagree, 2=Disagree, 3=Neither agree nor disagree, 4=Agree, 5=Strongly agree) - Corporate funding of academic research can create a conflict of interest (1=Strongly disagree, 2=Disagree, 3=Neither agree nor disagree, 4=Agree, 5=Strongly agree) - The risks to researchers of accepting funding from corporations are exaggerated (1=Strongly disagree, 2=Disagree, 3=Neither agree nor disagree, 4=Agree, 5=Strongly agree) - Corporate funding of academic research can create risks of undue influence on the research process (1=Strongly disagree, 2=Disagree, 3=Neither agree nor disagree, 4=Agree, 5=Strongly agree) - Corporate funding of academic research can compromise academic freedom (1=Strongly disagree, 2=Disagree, 3=Neither agree nor disagree, 4=Agree, 5=Strongly agree) - Universities should develop policies to protect research integrity from corporate influence (1=Strongly disagree, 2=Disagree, 3=Neither agree nor disagree, 4=Agree, 5=Strongly agree) - I am aware of the policies and procedure the University of Bath has in place to monitor and manage conflicts of interest in research (1=Strongly disagree, 2=Disagree, 3=Neither agree nor disagree, 4=Agree, 5=Strongly agree) - I would know how to manage the risks of corporate funding (1=Strongly disagree, 2=Disagree, 3=Neither agree nor disagree, 4=Agree, 5=Strongly agree) - I feel confident that I know how to mitigate the risks of corporate funding (1=Strongly disagree, 2=Disagree, 3=Neither agree nor disagree, 4=Agree, 5=Strongly agree)   Practice:   - I have applied the knowledge created in this course to my work. (1=Strongly disagree, 2=Disagree, 3=Neither agree nor disagree, 4=Agree, 5=Strongly agree) - I have applied the tools learned during the course in practice. (1=Strongly disagree, 2=Disagree, 3=Neither agree nor disagree, 4=Agree, 5=Strongly agree) - The training has had a positive impact on my research practices ((1=Strongly disagree, 2=Disagree, 3=Neither agree nor disagree, 4=Agree, 5=Strongly agree) - Have you done (or do you plan to do) anything new/different as a result of the training? (open question) - What factors helped you use the content of this course in your work? (Select all that apply: Time, support from colleagues, support from my supervisor, support from the University, opportunities to apply skills in a practical setting, Other (please specify)) - What factors kept you from using the content of this course in your work? (Select all that apply: Time, I did not remember the course content well enough to use it, lack of support from colleagues, lack of support from my supervisor, lack of support from the University, I did not have opportunities to apply skills in a practical setting, The course content was not relevant to my work, Other (please specify)) - I would recommend the training to colleagues/students (1=Strongly disagree, 2=Disagree, 3=Neither agree nor disagree, 4=Agree, 5=Strongly agree) - Is there anything you would like to add? |
| --- |
