# Supplementary figures and images for: Developing and evaluating an educational intervention on conflicts of interest and corporate influence on science
Source: Health Promot Int. 2025 May 22;40(3):daaf059. doi: 10.1093/heapro/daaf059 (PMC12096445; doi:10.1093/heapro/daaf059)

**Supplementary File 3. Illustration of coding using examples**


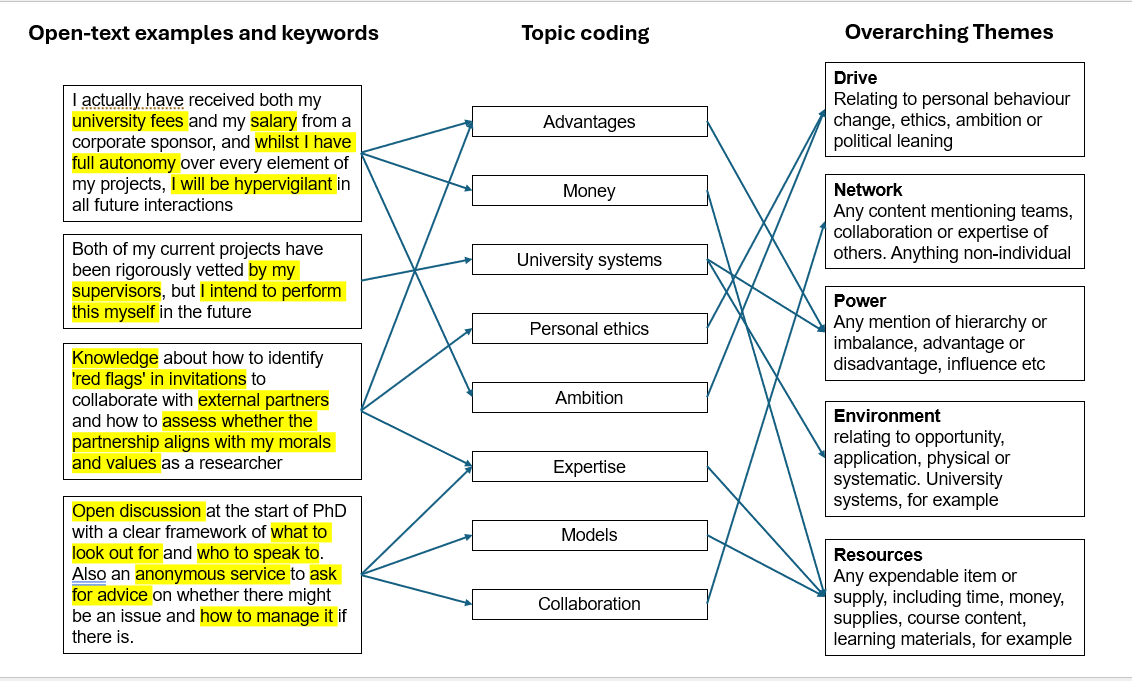

Supplement: daaf059_suppl_Supplementary_File_S3 [file daaf059_suppl_supplementary_file_s3.docx]
